# What factors predict physicians' utilization behavior of contrast-enhanced ultrasound? Evidence from the integration of the Theory of Planned Behavior and Technology Acceptance Model using a structural equation modeling approach

**DOI:** 10.1186/s12911-021-01540-8

**Published:** 2021-05-31

**Authors:** Qingwen Deng, Yuhang Zheng, Junhong Lu, Zhichao Zeng, Wenbin Liu

**Affiliations:** Department of Health Management, Room 108 in the Building for School of Public Health, Fujian Medical University, No. 1 Xuefubei Road, Minhou District, Fuzhou, 350122 China

**Keywords:** Technology adoption, Theory of planned behavior, Technology acceptance model, Contrast-enhanced ultrasound, Utilization

## Abstract

**Background:**

The promotion of early diagnosis is undoubtedly effective in reducing the burden of disease. Contrast-enhanced ultrasound (CEUS) is a diagnostic technology for liver cancer, but its implementation faces some challenges. Understanding the influencing factors of CEUS utilization is crucial for its successful implementation. However, such research is rare. The aims of this study were to investigate the status of CEUS utilization and its predictors in China.

**Methods:**

Through multistage random sampling, a cross-sectional study design was conducted among physicians in charge of direct use of CEUS working at liver disease-related departments of sampled health institutions. To access the potential influencing factors of physicians' CEUS utilization, a structured questionnaire was developed based on the theoretical model, which was developed by integration of the Theory of Planned Behavior (TPB) and Technology Acceptance Model (TAM). Structural equation modeling was used to verify the proposed hypotheses, and analyze the relationship and mechanism between the factors.

**Results:**

A total of 309 physicians were enrolled. The mean score of utilization behavior was 2.04 (SD = 1.07), and 37.22% above the mean. The favorable fitting results demonstrated that the integration of TAM and TPB was an acceptable model. SEM results also identified physicians’ intentions to use CEUS was directly associated with utilization behavior (*β* = 0.287, *P* < 0.001). Attitude (*β* = 0.272, *P* < 0.001), subjective norm (*β* = 0.172, *P* = 0.013), perceived behavioral control (*β* = 0.491, *P* < 0.001) and perceived usefulness (*β* = 0.108, *P* = 0.027) significantly influenced physicians’ intentions. Besides, subjective norm (*β* = 0.065, *P* = 0.021), perceived behavioral control (*β* = 0.141, *P* = 0.003), and perceived ease of use (*β* = 0.022, *P* = 0.033) indirectly affected physicians’ CEUS utilization.

**Conclusions:**

The findings provide a reference for understanding the factors associated with physicians' utilization of CEUS. Additionally, the proposed measures such as building innovative and incentive environment, providing high quality and adequate training, etc., will help promote the utilization of CEUS, thereby increasing the detection rate of liver cancer, and improving the survival rate and the quality of life for liver cancer patients.

**Supplementary Information:**

The online version contains supplementary material available at 10.1186/s12911-021-01540-8.

## Background

Over the last decades, the incidence of liver cancer has increased worldwide. In China, liver cancer is the second and third leading cause of cancer death in males and females [1], which remains a heavy disease burden for the individual, family and societal level [2]. Clinical practices indicate that once the symptoms of liver cancer have been discovered, most of the cases already have entered the advanced stage with an extremely low survival rate [3]. For the urgent demand of extending life expectance and improving quality of life, the importance of implementing effective technologies for early detection and diagnosis of liver cancer is becoming increasingly prominent [4]. Comparing with traditional ultrasonography [5, 6], contrast-enhanced ultrasound (CEUS) is one of the confirmed effective technologies with higher sensitivity and specificity in liver cancer diagnosis [7]. However, its implementation still faces some challenges and its current use is mainly limited to large urban hospitals [8]. Given the effectiveness of CEUS, it is necessary to fully understand its utilization status and potential influencing factors, so as to give full play to its expected benefits.

Since the individual-level and technology-level factors were generally considered as the two most critical factors [9, 10], some classical theories or theoretical models had been put forward to investigate technology utilization and implementation from a certain perspective, such as the Theory of Planned Behavior (TPB) and Technology Acceptance Model (TAM) [11–15]. In TPB, some individual-level factors have been demonstrated as potential influence factors on the final behavior. It suggests that a person's final behavior is driven by behavioral intention, while the behavioral intention is a function of three elements: attitude, subjective norm and perceived behavioral control [16]. Attitude is an individual's positive or negative evaluation of certain behavior. Subjective norm refers to the social pressure (from people who influence individuals, such as superiors and colleagues) that individuals feel on whether to take a particular behavior. Perceived behavioral control is the perception of the controllable degree to contributors and obstacles to take a particular behavior. TAM is also one of the most cited typical theoretical models in the field of technology adoption [14, 15], which mainly includes technology-level factors. According to TAM, perceived usefulness and perceived ease of use are two crucial aspects, which refer to the technical attributes influencing technology adoption or implementation [17]. Perceived usefulness is a degree to which a technology is believed to improve performance, while perceived ease of use reflects the degree to which technology can be easily used.

On the basis of TPB or TAM, several studies have demonstrated their explanatory power in interpreting specific behaviors of health professionals, such as compliance with guidelines, adoption of health information technology, etc. [18–21]. The association between final technology utilization behavior and individual-level (or technology-level) factors was verified [22, 23], which highlighted the great importance of the potential influencing factors at these two levels. However, to the best of our knowledge, few studies investigated the physicians' utilization behavior of CEUS integrating both the individual and technology levels. Additionally, the analytical methods used in previous research, such as descriptive analysis, difference comparison, linear regression analysis, was unable to simultaneously determine the relationship between different factors within the mechanism and test the potential mediating effects.

Therefore, this study aims to investigate physicians' CEUS utilization behavior and its predictors from both individual and technology levels by integrating TPB and TAM, and to examine the overall mechanism through the structural equation model (SEM). The findings will bridge the knowledge gap of factors associated with physicians' CEUS utilization behavior and provide references for expanding the implementation of CEUS and other appropriate health services or products.

## Theoretical model and hypotheses

**Fig. 1:**
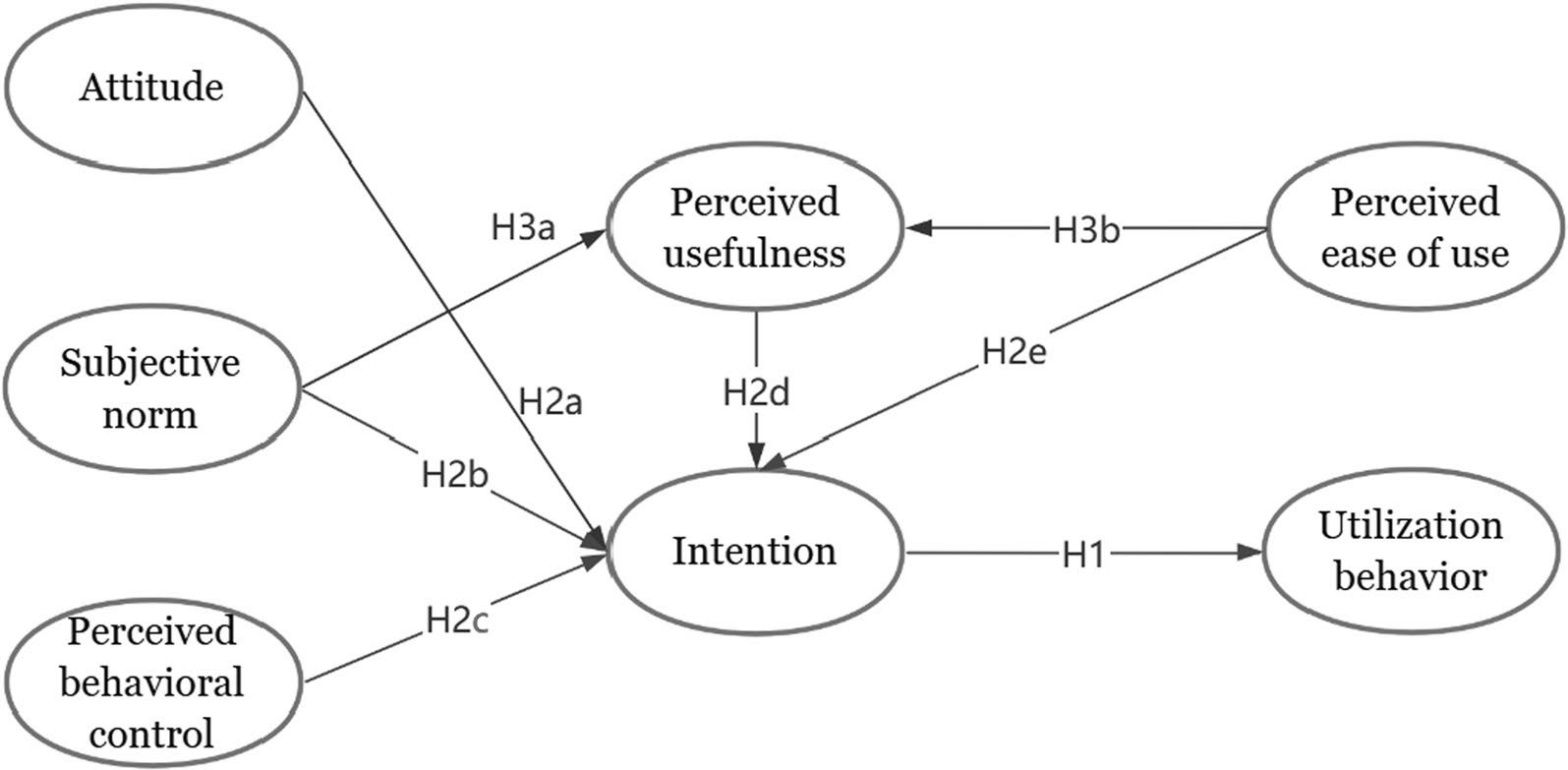
The theoretical model

To comprehensively investigate the mechanism on the utilization of CEUS of physicians from the levels of individual and technology, the theoretical model of this study was inspired by the integration of the TPB and TAM, which took seven elements into account, namely attitude, subjective norm, perceived behavioral control, perceived usefulness, perceived ease of use, behavioral intention, and final utilization behavior. The proposed theoretical model is presented in Fig. 1, and the corresponding hypothesis (H) is as follows:

### H1

Physicians' utilization behavior is influenced by their intentions to use CEUS.

### H2a

Physicians' intentions to use CEUS is influenced by their attitude.

### H2b

Physicians' intentions to use CEUS is influenced by their subjective norm.

### H2c

Physicians' intentions to use CEUS is influenced by their perceived behavioral control.

### H2d

Physicians' intentions to use CEUS is influenced by their perceived usefulness.

### H2e

Physicians' intentions to use CEUS is influenced by the perceived ease of use.

### H3a

Physicians' perceived usefulness of CEUS is influenced by their subjective norm.

### H3b

Physicians' perceived usefulness of CEUS is influenced by their perceived ease of use.

## Methods

### Study setting and population

In China, health institutions are the main users of various health technologies. In recent years, almost all health institutions at different levels have been included in a set of integration models, namely medical consortium, which is mainly characterized as technical cooperation and assistance. Thus, this study was implemented in the health institutions capable of deploying CEUS in the context of medical consortiums, which often refer to county hospitals or higher levels. And the physicians in charge of direct use of CEUS working in such health institutions were taken as the target population.

### Sampling approach

A cross-sectional study design was conducted from February to August 2019. A multistage random sampling method was used to select the samples. Firstly, Fujian and Jiangxi were randomly selected from the provinces with a high and low incidence of liver cancer, respectively. Secondly, two medical consortiums were selected from each province, and health institutions capable of deploying CEUS within the medical consortiums were randomly selected to include in the survey according to the proportion of 50%. Thirdly, all physicians who had knowledge of CEUS and worked at liver disease-related departments (such as the department of hepatology, oncology, gastroenterology, infection, ultrasound, etc.) would be invited to participate in this study. It was expected that 5 ~ 8 health institutions qualified to deploy CEUS would be investigated in each medical consortium. About 20 ~ 30 health institutions would be included in the four medical consortiums of two provinces. Since an average of 10 ~ 20 physicians would be sampled in each health institution, at least 200 physicians would participate in the survey, which can fully meet the basic requirement that the sample size should be set at least five times survey question [24].

### Data collection

Data were collected through a structured self-administered questionnaire, which was based on the integration of the TPB and TAM. With the support of selected health institutions, each round for filling out the questionnaire was accompanied by a trained coordinator to introduce the study purpose. Participation in the study was voluntary, and all responses were anonymous, but participants were invited to submit their contact information if they were interested in this study or wanted to be kept informed of the study results. Ethical permission was granted for this study from the Ethics Committee of Fujian Medical University (No. 2017-17).

### Measurements

This study included the utilization behavior of CEUS as an outcome variable. Additionally, six predictors of intention, attitude, subjective norm, perceived behavioral control, perceived usefulness, and perceived ease of use were also included. The utilization behavior of CEUS was measured by five items derived from the Knott and Wildavsky scale [25], which represents the five stages of utilization behavior: reference, effort, adoption, implementation, and impact. Variables of intention, attitude, subjective norm, and perceived behavioral control were all measured by three items derived from the TPB scale. Similarly, variables of perceived usefulness and perceived ease of use were measured by three items derived from the TAM scale. Each item was rated on a five-point Likert scale ranging from 1 (strongly disagree) to 5 (strongly agree). Besides, demographic information, such as gender, age, education level, professional title, administration position, and years in practice, were also collected. The final version of the questionnaire can be found in Additional file 1.

### Data analysis

Invalid questionnaires, including those that were incomplete or provided the same response for all items or with many missing values, were eliminated. SPSS 21.0 and AMOS 17.1 software programs were used as the two main statistical tools to analyze the data. Descriptive analyses were performed to illustrate the participants' demographic characteristics and the magnitude of CEUS utilization and its predictors. Besides, SEM was conducted to verify the proposed hypotheses and determine the mechanism. The *χ*^2^/df ratio, comparative fit index (CFI), Tucker-Lewis Index (TLI), and root mean square error of approximation (RMSEA) were used for checking the fitness of the model. *P* values less than 0.05 were considered significant in all reported analyses.

### Instrument reliability and validity

Table 1 shows the Cronbach’s alpha of each construct and the whole questionnaire are greater than the recommended threshold of 0.7. Besides, all corrected item-total correlation values were higher than the acceptability value of 0.5, suggesting the internal consistency of the questionnaire was fairly well.

**Table 1:** Reliability test for each item, variable and the whole questionnaire

Variable/item	Corrected item-total correlation	Cronbach’s α if item deleted	Cronbach’s α
*Attitude (ATT)*			0.921
ATT1	0.835	0.890	
ATT2	0.860	0.870	
ATT3	0.825	0.898	
*Subjective norm (SN)*			0.951
SN1	0.886	0.936	
SN2	0.922	0.908	
SN3	0.884	0.938	
*Perceived behavioral control (PBC)*			0.948
PBC1	0.867	0.942	
PBC2	0.919	0.903	
PBC3	0.889	0.926	
*Perceived usefulness (PU)*			0.936
PU1	0.866	0.910	
PU2	0.874	0.904	
PU3	0.866	0.910	
*Perceived ease of use (PEOU)*			0.852
PEOU1	0.725	0.792	
PEOU2	0.723	0.797	
PEOU3	0.726	0.792	
*Behavioral intention (BI)*			0.951
BI1	0.897	0.927	
BI2	0.885	0.936	
BI3	0.906	0.920	
*Utilization behavior (UB)*			0.944
UB1	0.856	0.929	
UB2	0.823	0.935	
UB3	0.793	0.940	
UB4	0.878	0.925	
UB5	0.888	0.923	
The whole questionnaire			0.947

Regarding the validity, the Kaiser–Meyer–Olkin (KMO) and Bartlett's test of sphericity were applied to the first-step analysis. The results showed that the KMO value of 0.948, and Bartlett's test of sphericity was strongly significant (*P* < 0.001), indicating the great suitability of this instrument for validity estimate. To further validate the instrument, we calculated four commonly used indicators to assess convergent validity and discriminant validity: factor loading of each item, average variance extracted (AVE), composite reliability (CR), and the square root of AVE for each construct. The results showed the value of these four indicators were above the recommended value of 0.5, 0.5, 0.7 and 0.7, respectively (Tables 2 and 3), which indicates an acceptable convergent and discriminant validity.

**Table 2:** Convergent validity test

Variable	Item	Factor loading	AVE	CR
Attitude	ATT1	0.905	0.863	0.950
	ATT2	0.944		
	ATT3	0.938		
Subjective norm	SN1	0.943	0.912	0.969
	SN2	0.968		
	SN3	0.954		
Perceived behavioral control	PBC1	0.934	0.902	0.965
	PBC2	0.963		
	PBC3	0.952		
Relative advantage	RA1	0.880	0.801	0.924
	RA2	0.903		
	RA3	0.902		
Perceived ease of use	PEU1	0.848	0.657	0.851
	PEU2	0.764		
	PEU3	0.817		
Behavioral intention	BI1	0.965	0.894	0.962
	BI2	0.930		
	BI3	0.942		
Utilization behavior	UB1	0.650	0.718	0.933
	UB2	0.829		
	UB3	0.849		
	UB4	0.939		
	UB5	0.936

**Table 3:** Discriminant validity test

Variable	Attitude	Subjective norm	Perceived behavioral control	Relative advantage	Perceived ease of use	Behavioral intention	Utilization behavior
Attitude	**0.929**						
Subjective norm	0.925	**0.955**					
Perceived behavioral control	0.919	0.885	**0.950**				
Relative advantage	0.471	0.510	0.451	**0.895**			
Perceived ease of use	0.000	0.000	0.691	0.691	**0.810**		
Behavioral intention	0.923	0.914	0.942	0.547	0.076	**0.946**	
Utilization behavior	0.268	0.262	0.270	0.268	0.022	0.289	**0.847**

Bold values in the diagonal indicate the square root of AVE of the corresponding variable

## Results

### Participant characteristics

A total of 329 questionnaires were distributed. After excluding invalid questionnaires, 309 were included for the analysis, with a valid response rate of 93.92%. The demographic characteristics of the participants are shown in Table 4. Among the participants, 65.05% were males, 48.54% were below 35 years old, 91.26% had a bachelor's degree or above, 42.39% had obtained intermediate professional title, 82.85% had no administration position, and 64.40% had 5 to 15 years of practice.

**Table 4:** Demographic characteristics of the participants (n = 309)

Characteristics	Frequency	Percentage
*Gender*		
Male	201	65.05
Female	108	34.95
*Age*		
< 35 years old	150	48.54
35 ~ 44 years old	115	37.22
≥ 45 years old	44	14.24
*Education level*		
Junior college or less	27	8.74
Bachelor	172	55.66
Master or above	110	35.60
*Professional title*		
Junior	106	34.30
Intermediate	131	42.39
Senior	72	23.30
*Administration position*		
No	256	82.85
Yes	53	17.15
*Years in practice*		
< 5 years	72	23.30
5 ~ 15 years	199	64.40
> 15 years	38	12.30

### Measurement scores of all predictors and utilization behavior of CEUS

The mean score of utilization behavior was 2.04, and 37.22% scored above the mean value. Attitude, subjective norm, perceived behavioral control, perceived usefulness, perceived ease of use, and intention had a mean score of 4.28, 4.18, 4.27, 4.36, 4.20, and 4.31, respectively (Table 5). The proportion of the participants scored above the average value of variable scores was ranged from 45.63 to 51.78%.

**Table 5:** Measurement scores of participants

Measurements	Mean	Standard deviation	Median	N (%) of scores > mean
Attitude	4.28	0.78	4.33	160 (51.78)
Subjective norm	4.18	0.83	4.00	141 (45.63)
Perceived behavioral control	4.27	0.77	4.00	153 (49.51)
Perceived usefulness	4.36	0.67	4.33	148 (47.90)
Perceived ease of use	4.20	0.71	4.00	140 (45.31)
Behavioral intention	4.31	0.78	4.33	159 (51.46)
utilization behavior	2.04	1.17	1.60	115 (37.22)

### Tests of model and hypotheses

A favorable fitness of data into the theoretical model was found: χ^2^/df = 2.906 (< 5), CFI = 0.947 (> 0.9), TLI = 0.939 (> 0.9), and RMSEA = 0.079 (< 0.08).

**Fig. 2:**
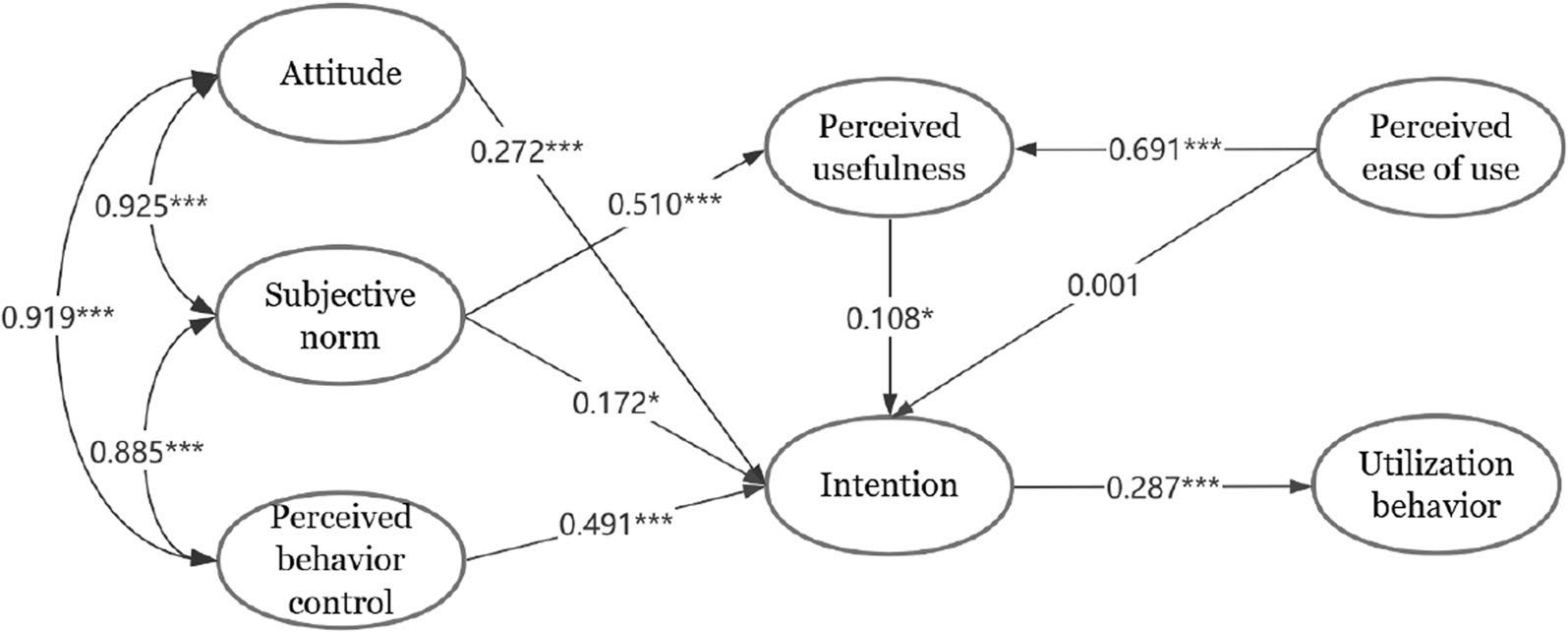
Model of utilization behavior of CEUS among 309 physicians (*P < 0.05; *** *P* < 0.001). *Notes*: the figures placed in the lines with an arrowhead between the variables represent standardized regression coefficients, i.e. factor loading

After the measurement model was confirmed, we used a structural model to verify the proposed research hypotheses. The final structural model with the estimated standardized coefficients is presented in Fig. 2, and the estimation results of the hypotheses are shown in Table 6. In addition to perceived ease of use (*β* = 0.001, *P* = 0.975), attitude, subjective norm, perceived behavioral control and perceived usefulness were found to have significant effects on behavioral intention (*P* < 0.05). Moreover, utilization behavior was significantly influenced by behavioral intention (*β* = 0.287, *P* < 0.001).

**Table 6:** Results of structural equation modeling analysis

The hypothesis (H)	Factor loading	Standard error	Critical ratio	*P* value	Support
H1: Utilization behavior ← Intention	0.287	0.087	4.998	< 0.001	Yes
H2a: Intention ← Attitude	0.272	0.071	3.349	< 0.001	Yes
H2b: Intention ← Subjective norm	0.172	0.060	2.491	0.013	Yes
H2c: Intention ← Perceived behavioral control	0.491	0.054	7.881	< 0.001	Yes
H2d: Intention ← Perceived usefulness	0.108	0.073	2.219	0.027	Yes
H2e: Intention ← Perceived ease of use	0.001	0.037	0.032	0.975	No
H3a: Perceived usefulness ← Subjective norm	0.510	0.024	12.600	< 0.001	Yes
H3b: Perceived usefulness ← Perceived ease of use	0.691	0.029	13.798	< 0.001	Yes

### Tests of indirect effects

Indirect effects were also assessed in this study. The results of the Bootstrap test showed that the indirect effects of subjective norm, perceived behavioral control, and perceived ease of use on physicians' utilization behavior of CEUS was 0.065 (*P* = 0.021), 0.141 (*P* = 0.003), and 0.022 (*P* = 0.033), respectively. On the contrary, the indirect effects of attitude (*P* = 0.107) and perceived usefulness (*P* = 0.071) on physicians' utilization behavior of CEUS were not found.

## Discussion

To bridge the knowledge gap of the CEUS utilization behavior and its influencing factors among physicians in China, this study put forward the theoretical model by integrating TPB and TAM, which comprehensively took the two crucial sets of factors at individual-level and technology-level into account. Additionally, to make the data analysis more accurate and robust, the SEM approach was performed to simultaneously investigate the influencing factors of CEUS utilization behavior, as well as the internal interaction between these factors. Our findings revealed that physicians' utilization behavior of CEUS depended on their behavioral intentions to CEUS, while their intentions were directly influenced by multiple factors, including individual-level and technology-level factors. All but one hypothesis (H2e) succeed validation in our extended model, confirming the integration of TPB and TAM was an acceptable theoretical foundation for this study.

Consistent with TPB hypotheses and previous studies [26, 27], attitude, subjective norm and perceived behavior control were the important predictors of technology adoption in health care professionals [28]. It appeared that the use of CEUS often occurs to physicians who had a positive attitude toward CEUS, perceived the expectations of their colleagues and supervisors, or perceived the possible financial and reputation returns of using CEUS [29, 30]. These results demonstrated the important role of physicians in expanding technology utilization. And for CEUS in this study, more importance can be stressed on taking measures to promote physicians' positive evaluation of CEUS and its use.

The significant effect of perceived ease of use on the perceived usefulness of CEUS was confirmed in this study, as well as the impact of perceived usefulness on physicians' intention to use CEUS. These results were also shown in many previous studies that in clinical practice, physicians usually attach considerable importance to the usefulness while adopting particular technology [31] to make work more efficient and effective. And the physicians tend to appreciate the usefulness of certain technology if it takes no extra time and effort to master it. Additionally, in line with the findings of previous studies in the field of health technology adoption [32, 33], this study also reported a significant relationship between subjective norm and perceived usefulness of CEUS, which can be described by an interesting term "internalization" [34] that physicians generally perceive the usefulness of certain technology when it is seen as useful by influential experts or other important persons [20]. These results not only confirmed the important role of individual-level factors in expanding CEUS utilization as mentioned above, but also highlighted the importance of technology itself. Technologies with greater efficacy and practicality tend to be adopted and used more widely. Besides, this study also reminds us of the key persons' demonstration effect during the process of technology utilization and diffusion.

It's worth noting that the significant relationship between perceived ease of use and physicians' intention to use CEUS was not found, failing to support Hypothesis 2e. Although many studies found that health professional's perceived ease of use was associated with their intention to use certain technology or product [35, 36], a study on emergency physicians reported that perceived ease of use can not increase the usage of computerized physician order entry system [37]. Another study conducted in the USA also found that the influence of perceived ease of use on pediatricians' intention to use internet-based health applications was not significant [38]. A plausible reason is that the effect of perceived ease of use on the usage intention will become non-significant with long-term exposure to the technology [20]. Another explanation is that the effect of perceived ease of use may be weakened as physicians with a high level of competency [39]. Although the influence of perceived ease of use on behavioral intention was insignificant, the indirect effects of perceived ease of use on physicians' utilization behavior of CEUS have been found. Therefore, the influence of perceived ease of use still cannot be ignored.

### Implications for practice

CEUS has been explored for diagnosis of hepatopathy for many years worldwide. Although it has similar performance to contrast-enhanced computed tomography (CT) and magnetic resonance imaging (MRI) in the demonstration of liver lesions and peripheral liver blood flow [40], CEUS has its unique advantages in certain regards. Contrast agents for CEUS are biodegradable microbubbles without nephrotoxicity, which can be safely used in patients contraindicated to CT/MRI contrast agents [41]. Compared with CT or MRI’s missed detection due to the mistiming of the arterial phase, CEUS is more sensitive by virtue of its dynamic real-time imaging. Moreover, CEUS has the strength of showing rapidly changing arterial phase enhancement patterns [41]. By comparison, CT and MRI often fail to reveal these rapidly changing features, which may cause diagnostic confusion [40]. It is precisely because of these outstanding features of CEUS that promoting the utilization of CEUS will have important significance for clinical practice, and the findings of the study have several implications for how to promote the utilization of CEUS.

Additionally, the results will inform hospital managers about the importance of establishing a favorable impression of certain health technology and its utilization, so that a positive attitude can be developed. First, it's desirable for hospital managers to build a better innovative and incentive environment in which using innovative technology is an acceptable and even encouraged way to test liver cancer. Bulletin boards, cultural and creative products, and typical event learning are widely accepted forms. Through these forms, the emphasis given to the utilization of health technology can be increased to a certain extent, thereby driving the development of a good atmosphere. Second, for expanding the utilization of health technology, senior physicians and managers who have a demonstration effect on most physicians should also be mobilized. For example, through some mentor-apprentice teaching and experience-sharing sessions, gradually adjust other physicians' compliance to the utilization of certain health technology. Third, physicians need to get high-quality and adequate training before using certain technology, so as they can gain more perception of its usefulness and ease of use [38]. In order to ensure the effectiveness of training, in addition to the reasonable arrangement of physicians' work and rest time, attention must also be paid to the combination of theory and practice. Each training scale should be mainly small or medium, and it needs to be equipped with multimedia audio-visual equipment and related operating equipment. Making concerted efforts mentioned above will help physicians develop social norms and beliefs about technology usage and attract more physicians to use CEUS.

### Strengths and limitations

In addition to implications, this study was also strengthened by some interesting features. One of the strengths was the use of an extended theoretical model, which was on the basis of integrated insights from the TPB and TAM. This has been shown to be useful in identifying predictors (individual- and technology-level) of physicians' CEUS utilization behavior and providing approaches for possible intervention. Secondly, the application of SEM made the result prediction more accurate, and the influence of several factors on the outcome variable and their internal interactions can be analyzed simultaneously. Thirdly, the sample areas included Fujian and Jiangxi provinces with a high and low incidence of liver cancer, respectively, which would benefit the representativeness of the samples.

However, there are some limitations to this study. First, since variables were measured by self-reports, we cannot ignore the possibility that the participants tend to make a positive response. Besides, further research can take more relevant factors into considerations, such as physicians expertise and patient dependency. Finally, due to limited time and funds, this study only included two sample areas, and it is recommended for future research to include more samples from different areas to make the results more generalizable.

## Conclusions

This study advanced knowledge about the predictors of physicians' CEUS utilization behavior based on the integration of TPB and TAM. In this regard, if the suggestive measures proposed in this study for hospital managers can be implemented, it will shape a tangible or intangible internalization impact on the clinical practice of CEUS for front-line physicians. When making technical decisions between CEUS and other diagnostic methods, physicians will draw on their perceptions about CEUS to support their choices, which include their attitude towards CEUS, ease of use and usefulness of CEUS, evaluation of CEUS by people around, and potential benefits of using CEUS, etc. In theory, the findings will provide a theoretical reference for future research on technology utilization and better understand the influencing factors of CEUS utilization. In practice, this is conducive to promote the utilization of CEUS, so as to improve the survival probability and quality of life for liver cancer patients, and reduce the burden of disease on the health system and patients’ families.

## Supplementary Information

**Additional file 1. Survey questionnaire.** (The instrument for this study to investigate the individual- and technology-level factors of CEUS utilization among physicians in China).

## Data Availability

The datasets generated during and/or analyzed during the current study are available from the corresponding author on reasonable request.

## Abbreviations

CEUS: Contrast-enhanced ultrasound
TPB: Theory of Planned Behavior
TAM: Technology Acceptance Model
H: Hypothesis
SD: Standard deviation
CT: Computed tomography
MRI: Magnetic resonance imaging
