# Supplementary material for: What factors predict physicians' utilization behavior of contrast-enhanced ultrasound? Evidence from the integration of the Theory of Planned Behavior and Technology Acceptance Model using a structural equation modeling approach
Source: BMC Med Inform Decis Mak. 2021 May 31;21:173. doi: 10.1186/s12911-021-01540-8 (PMC8165765; doi:10.1186/s12911-021-01540-8)
Supplement: Supplementary file 1 — Additional file 1. Survey questionnaire. (The instrument for this study to investigate the individual- and technology-level factors of CEUS utilization among physicians in China). [file 12911_2021_1540_MOESM1_ESM.doc]

**Research questionnaire of the individual- and technology-level factors**

**of CEUS utilization among physicians in China**

**Part 1. Structural Scales**

Notes. There are 5 numbers (1, 2, 3, 4, 5) on the right side of each item, where “1” means “Strongly disagree”, “2” means “Disagree”, “3” means “Neutral”, “4” means “Agree”, and “5” means “Strongly agree”. Please tick or circle the number that best fits your real feelings on the item.

| ***Attitude*** | | | | | |
| --- | --- | --- | --- | --- | --- |
| I think it’s a right thing to use CEUS for early diagnosis of hepatocellular carcinoma. | 1 | 2 | 3 | 4 | 5 |
| I think it’s a wise choice to use CEUS for early diagnosis of hepatocellular carcinoma. | 1 | 2 | 3 | 4 | 5 |
| I think it’s good for all to use CEUS for early diagnosis of hepatocellular carcinoma. | 1 | 2 | 3 | 4 | 5 |
| ***Subjective norm*** | | | | | |
| People who are important to me tend to use CEUS for early diagnosis of hepatocellular carcinoma. | 1 | 2 | 3 | 4 | 5 |
| People who are important to me have a positive attitude to use CEUS for early diagnosis of hepatocellular carcinoma. | 1 | 2 | 3 | 4 | 5 |
| People who are important to me think it’s a right thing to use CEUS for early diagnosis of hepatocellular carcinoma. | 1 | 2 | 3 | 4 | 5 |
| ***Perceived behavioral control*** | | | | | |
| Using CEUS can make me have more choice in diagnosing liver cancer. | 1 | 2 | 3 | 4 | 5 |
| Using CEUS can increase my confidence in diagnosing liver cancer. | 1 | 2 | 3 | 4 | 5 |
| Using CEUS can make my diagnosis more recognized. | 1 | 2 | 3 | 4 | 5 |
| ***Perceived usefulness*** | | | | | |
| The use of CEUS can reduce the rate of missed diagnosis and misdiagnosis of liver cancer. | 1 | 2 | 3 | 4 | 5 |
| The use of CEUS can improve the recognition of the diagnosis results of liver cancer. | 1 | 2 | 3 | 4 | 5 |
| The use of CEUS can improve the efficiency of diagnosing liver cancer. | 1 | 2 | 3 | 4 | 5 |
| ***Perceived ease of use*** | | | | | |
| We can easily obtain the materials and instruments needed for CEUS test. | 1 | 2 | 3 | 4 | 5 |
| We can get the result of CEUS test in a short time after detection. | 1 | 2 | 3 | 4 | 5 |
| We can be provided with assistance in clinical diagnosis by the result of CEUS test. | 1 | 2 | 3 | 4 | 5 |
| ***Behavioral intention*** | | | | | |
| I would like to use CEUS in the diagnosis of liver cancer. | 1 | 2 | 3 | 4 | 5 |
| I would like to learn skills and experience of using CEUS from my peers. | 1 | 2 | 3 | 4 | 5 |
| I would like to recommend the use of CEUS to my peers. | 1 | 2 | 3 | 4 | 5 |

| ***Utilization behavior*** | | | | | |
| --- | --- | --- | --- | --- | --- |
| In the past year, I took the initiative to search for information to learn how to use CEUS. | 1 | 2 | 3 | 4 | 5 |
| In the past year, I introduced the use of CEUS in the early diagnosis of liver cancer to patients. | 1 | 2 | 3 | 4 | 5 |
| In the past year, the probability that I use CEUS on all working days. | 1 | 2 | 3 | 4 | 5 |
| In the past year, the probability that I skillfully combine the CEUS results to make clinical diagnosis. | 1 | 2 | 3 | 4 | 5 |
| In the past year, the probability that I recommended further using CEUS to my peers. | 1 | 2 | 3 | 4 | 5 |

**Part 2. Personal Information Card**

1. Please choose your gender.

A. Male B. Female

2. Please write down your age: ________

3. Please choose your educational level.

A. Junior college or below B. Bachelor C. Master D. Doctor

4. Please choose your professional title.

A. Junior B. Intermediate C. Senior

5. Please choose your administration position.

A. Yes B. No

6. Please choose your years in practice.

A. <5 years B. 5~10 years C. 11~15years D. 16~20 years E. >20 years
